# Hornblendite delineates zones of mass transfer through the lower crust

**DOI:** 10.1038/srep31369

**Published:** 2016-08-22

**Authors:** Nathan R. Daczko, Sandra Piazolo, Uvana Meek, Catherine A. Stuart, Victoria Elliott

**Affiliations:** 1 ARC Centre of Excellence for Core to Crust Fluid Systems and GEMOC, Department of Earth and Planetary Sciences, Macquarie University, NSW 2109, Australia

## Abstract

Geochemical signatures throughout the layered Earth require significant mass transfer through the lower crust, yet geological pathways are under-recognized. Elongate bodies of basic to ultrabasic rocks are ubiquitous in exposures of the lower crust. Ultrabasic hornblendite bodies hosted within granulite facies gabbroic gneiss of the Pembroke Valley, Fiordland, New Zealand, are typical occurrences usually reported as igneous cumulate hornblendite. Their igneous features contrast with the metamorphic character of their host gabbroic gneiss. Both rock types have a common parent; field relationships are consistent with modification of host gabbroic gneiss into hornblendite. This precludes any interpretation involving cumulate processes in forming the hornblendite; these bodies are imposter cumulates. Instead, replacement of the host gabbroic gneiss formed hornblendite as a result of channeled high melt flux through the lower crust. High melt/rock ratios and disequilibrium between the migrating magma (granodiorite) and its host gabbroic gneiss induced dissolution (grain-scale magmatic assimilation) of gneiss and crystallization of mainly hornblende from the migrating magma. The extent of this reaction-replacement mechanism indicates that such hornblendite bodies delineate significant melt conduits. Accordingly, many of the ubiquitous basic to ultrabasic elongate bodies of the lower crust likely map the ‘missing’ mass transfer zones.

Earth has a heterogeneous, layered crust that overlies a relatively homogeneous mantle. Geochemical signatures in middle to upper crustal rocks suggest that they are sourced from melting of lower crustal and mantle environments^1^,^2^,^3^. This requires mass transfer through the lower crust. Though some geological pathways of magma migration are recognized as dykes^4^ and shear zones^5^, dykes are relatively rare and shear zones documented to be associated with mass transfer are insufficient to facilitate the volume required for crustal differentiation throughout Earth’s history^6^. Therefore, there must exist structures that are yet to be recognized as zones of substantial mass transfer. Elongate bodies of basic to ultrabasic rocks are ubiquitous in exposures of the lower crust. Hornblendite bodies of the Pembroke Valley, New Zealand, contain igneous features that contrast with the metamorphic character of their host gneiss. The field relationships are inconsistent with a cumulate origin for the hornblendite. In contrast, a model is presented in which melt flux and extreme melt-rock interaction results in modification of the host gneiss into hornblendite. We define our use of (i) ‘hydrous silicate melt’, hereafter referred to as melt, as a silicate magma with a H_2_O content at or below water saturation for given P-T-X conditions; (ii) ‘dissolution’ as grain-scale magmatic assimilation, analogous to assimilation of a xenolith in a magma chamber and distinct to *in situ* partial melting; and (iii) ‘flux’ as the passage of melt through the rock, distinct to ‘influx’ which implies injection and stagnation of melt.

## Results

**Figure 1:**
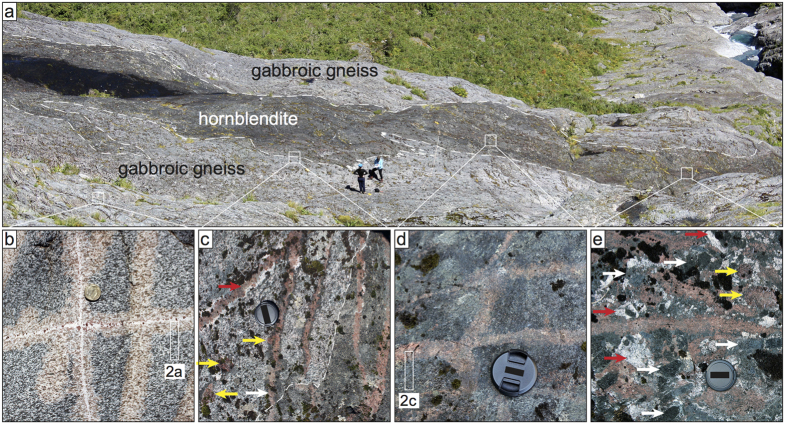
Relationships between the hornblendite and its host gneiss. (**a**) 30–40 m wide hornblendite body (dark rock outlined by dashed lines). Locations of detailed photographs in (**b–e**) shown by small squares. (**b**) Host gneiss with rectilinear grid pattern of garnet granulite reaction zones. Location of photomicrograph in 2a shown. (**c**) Sharp contact (dashed line) between hornblendite and a transition zone characterized by garnet surrounded by thin leucosome (yellow arrows) and garnet trains surrounded by plagioclase (red arrow) or hornblende (white arrow). (**d**) Grid pattern of garnetite stringers in hornblendite body. Location of photomicrograph in 2c shown. (**e**) Pegmatitic coarse euhedral garnet (yellow arrows), plagioclase (red arrows) and hornblende (white arrows) within hornblendite. Note finer-grained garnetite stringer (left to right, center) and clinozoisite-rich hornblendite matrix to coarser-grained pegmatitic minerals.

**Figure 2:**
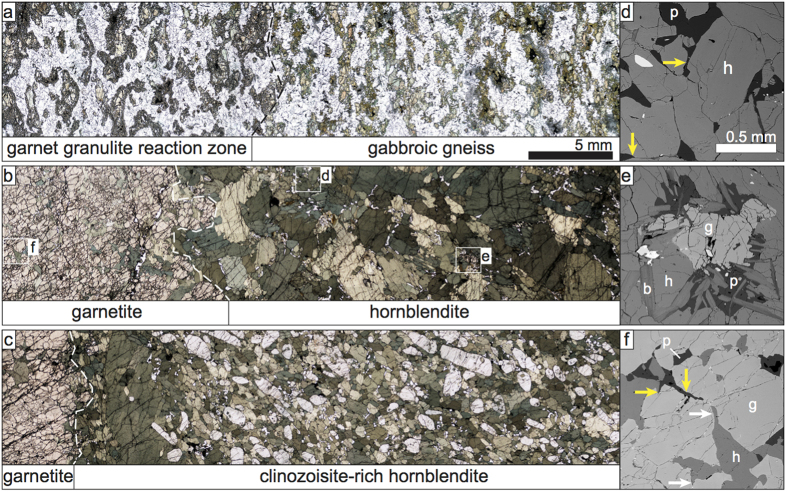
Petrographic relationships within the hornblendite and its host gneiss. (**a**) Two-pyroxene-hornblende gneiss (right) and garnet granulite reaction zone (left). (**b**) Hornblendite (right) and garnetite stringer (left). (**c**) Clinozoisite-rich hornblendite (right) and garnetite stringer (left). Note shape preferred orientation of elongate clinozoisite and hornblende grains. (**d**–**f**) Back-scattered electron images of low dihedral angles, films along grain boundaries and small pockets representing the crystallization of former melt, along with well-developed crystal faces at unlike mineral boundaries (e.g. plagioclase-hornblende boundaries in upper left of **f**). Mineral labels are plagioclase (p, yellow arrows), hornblende (h, white arrows), biotite (b) and garnet (g).

Granulite facies two-pyroxene-hornblende gabbroic gneiss (pale rock, Fig. 1a), with ubiquitous garnet granulite reaction zones (Fig. 1b), is the main rock type exposed^7^,^8^,^9^. The gneissosity is defined by elongate clusters of pyroxene and hornblende within a plagioclase matrix. All minerals display evidence of dynamic recrystallisation including undulose extinction, presence of subgrains and a bimodal grain size distribution. Reaction zones are directly associated with felsic dykes which cut the gabbroic gneiss forming a distinct rectilinear grid pattern (Fig. 1b). In reaction zones, garnet grains partially to completely pseudomorph the gneissosity (Fig. 2a)^7^,^8^. This grid pattern of garnet granulite reaction zones provides unique markers. Reaction zones may be traced from the gabbroic gneiss (Fig. 1b) across a narrow (2–7 m wide) transition zone (Fig. 1c) into 30–40 m wide bodies of hornblendite (Fig. 1c,d) that are hosted by the gabbroic gneiss (Fig. 1a). The garnet granulite reaction zones change along strike into garnet trains variably surrounded by plagioclase (red arrow, Fig. 1c) or hornblende (white arrow, Fig. 1c) and are continuous with garnetite stringers (<5 cm wide) in the hornblendite (Fig. 1c,d).

Hornblendite bodies have highly irregular boundaries (dark rock, Fig. 1a), and may contain patches rich in clinozoisite (Fig. 1c,d) and/or large euhedral garnet (<10 cm across). Within the hornblendite, stringers of garnetite occur in a grid pattern (Fig. 1d) closely resembling the pattern made by garnet granulite reaction zones in the gabbroic gneiss. Garnetite stringers are locally folded and/or dismembered. Metre-scale areas of pegmatitic plagioclase-hornblende-garnet occur within the hornblendite with euhedral mineral grain sizes up to 10 cm (Fig. 1e).

The hornblendite (grains <5 mm; Fig. 2b) may contain up to 20 vol.% prismatic clinozoisite (<5 mm long; Fig. 2c). Both minerals exhibit unimodal grain size (Fig. 2b,c) and clinozoisite grains contain inclusions of hornblende. The unit contains minor interstitial plagioclase (~1 vol.%) with some dihedral angles <10° (Fig. 2d). Rare fine-grained biotite, hornblende, garnet and rutile occur in small plagioclase-rich domains (Fig. 2e). Grains in the hornblendite lack evidence of internal crystal plastic deformation, but may show a shape preferred orientation (Fig. 2c). Garnetite stringers in the hornblendite comprise >95 vol.% garnet (Fig. 2b,c,f). Within and directly adjacent to the garnetite stringers, minor interstitial plagioclase and hornblende may have dihedral angles <10° (Fig. 2f).

The narrow transition zone between the host gabbroic gneiss and hornblendite bodies is characterised by large garnet grains (<10 cm across) surrounded by thin plagioclase rims, i.e. leucosome consistent with limited *in situ* partial melting of the host gneiss (<0.5 cm across; yellow arrows, Fig. 1c). Across the transition zone, the gneissic foliation and garnet granulite reaction zones progressively change orientation to become subparallel to the boundary of the hornblendite bodies (Fig. 1c).

The continuity and progressive modification of the garnet granulite reaction zones in the host gabbroic gneiss, to garnetite stringers in the hornblendite, link the two contrasting rock types. In the following, the igneous character of the hornblendite bodies is contrasted with the metamorphic character of the host gabbroic gneiss.

## Discussion

The igneous nature of the hornblendite unit is supported by the following features: (i) high mode of one or two minerals (hornblende ± clinozoisite or garnet) with unimodal grain size distribution and interlocking euhedral grain shapes; (ii) interstitial minor phases (plagioclase) with low dihedral angles, representing pseudomorphs of former melt^10^,^11^; (iii) presence of domains exhibiting a shape preferred orientation of hornblende and clinozoisite grains while lacking microstructures typical of crystal plastic deformation; (iv) presence of small pockets rich in plagioclase and biotite, of approximate granodioritic composition^12^; and (v) minor pegmatitic domains with very coarse, randomly oriented, interlocking euhedral grains. These characteristics are not only typical for an igneous ultrabasic rock but are also consistent with a cumulate origin. In contrast, the gabbroic gneiss that hosts the hornblendite (Fig. 1a) has tectono-metamorphic features such as a gneissic foliation, evidence of crystal plastic deformation at the grain scale, and well-studied metamorphic reaction textures^8^,^13^,^14^,^15^.

The presence of a body with typical igneous character within a high-grade metamorphic gneiss, without a structural break, requires that the former is younger than its metamorphic host. The classic interpretation here would involve intrusion of the hornblendite into the metamorphic gneiss, for example as a dyke. However, this is inconsistent with the highly irregular boundaries of the hornblendite, and the mineral assemblage would require an unusual ultrabasic composition of the intruding body. Importantly, the physical continuity and progressive modification of metamorphic microstructures and assemblage of the garnet granulite reaction zones into the igneous microstructures and assemblage of the garnetite stringers precludes any interpretation involving dyke-like intrusion of the hornblendite bodies. Consequently, this focuses attention on the processes that can explain these intriguing relationships.

A viable option to explain both the field and petrographic relationships involves the modification (reaction replacement) of the host gabbroic gneiss to form hornblendite as a consequence of channeled high melt flux through the lower crust. In this case, the continuity of the pre-existing garnet granulite reaction zones and their modified counterparts (i.e. garnetite stringers) is explainable by extensive melt-rock interaction due to flux of an externally-derived hydrous silicate melt that is in disequilibrium with the gneiss it migrates through. Consequently, the hornblendite bodies represent “imposter cumulates” that delineate channels of mass transfer.

**Figure 3:**
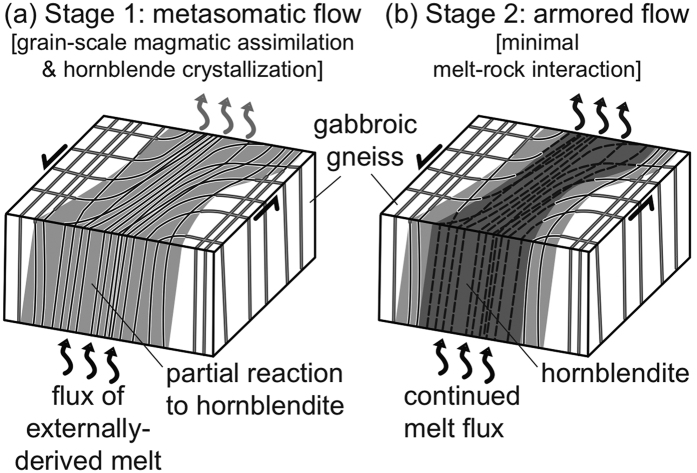
Schematic diagram showing the two stages involved in the production of hornblendite as the result of channeled, externally-derived melt flux through the lower crust. (**a**) Stage 1: gabbroic gneiss (white) with grid pattern of garnet granulite reaction zones is progressively modified by melt-rock reactions to become successively depleted in plagioclase and pyroxene through dissolution (grain-scale magmatic assimilation) and enriched in hornblende and clinozoisite by crystallization (light grey, **a**) to become hornblendite (dark grey, **b**). (**b**) Stage 2: mass transfer within channelized melt flux zone delineated by hornblendite; the zone is armored from chemical interaction with the host rock during continued melt flux.

A model is proposed, in which the gabbroic gneiss and garnet granulite reaction zones in the host rock are progressively replaced during melt-rock interaction by hornblendite (±clinozoisite) and garnetite, respectively. The interaction involves (i) flux of an externally-derived hydrous silicate melt, (ii) dissolution (grain-scale magmatic assimilation) of host gneiss plagioclase and pyroxene, and (iii) crystallization of hornblende (±clinozoisite) and garnet from the migrating magma during channelized melt flow (Fig. 3). Two main stages in the development of hornblendite bodies can be distinguished, encompassing (i) melt flux of an externally-derived hydrous silicate melt in a high-strain zone, causing chemical and mineral assemblage modification of the host rock (metasomatism) due to extensive melt-rock interaction, and (ii) armored channelized melt flux with little further interaction (Fig. 3). The partial to complete reaction-replacement of host gneiss plagioclase and pyroxene in the first stage closely resembles the production of hornblendite from clinopyroxenite cumulate rocks documented in other arc settings^16^, where clinopyroxene-melt reaction produces hornblende as later melts are inferred to have ascended through the cumulate pile. A similar dissolution mechanism is proposed here, whereby hydrous silicate melts capable of precipitating hornblende (±clinozoisite) and garnet are fluxed through 30–40 m wide zones of lower crust (Fig. 3, stage 1). A reactive infiltration instability process is invoked, where one or more phases (plagioclase and pyroxene in this case) dissolve to enhance porosity and permeability, which enhances flow of the externally-derived hydrous silicate melt, and focuses porous flow within the channel^17^,^18^,^19^.

The localized strain observed within and at the margins of the hornblendite bodies suggest porous melt flow was facilitated and/or enhanced by localized deformation^20^,^21^,^22^,^23^ which also increases the porosity and permeability of the melt flux zone. As the high melt flux event progresses, the minerals within the host rock that are in disequilibrium with the melt are completely dissolved via grain-scale magmatic assimilation. The crystallization of new phases (mainly hornblende) from the migrating melt locally produces an unreactive conduit. As a result, the channel becomes chemically armored and a large volume of melt can be transported through the channel without any further physical or chemical fingerprint (Fig. 3, Stage 2). Similar chemically isolated or armored conduits, where little to no reaction occurs between the migrating magma and surrounding rock, have been identified in a less hydrous and more mafic system in the mantle^17^,^18^,^19^,^24^. For this scenario, migration processes are well documented in experiments^25^,^26^ and numerical models^27^,^28^,^29^. As our system wanes and the conduit cools, final crystallization produces minerals with small dihedral angles (Fig. 2d,f) and pockets of melt crystallize to form fine-grained biotite, hornblende, garnet and rutile in small plagioclase-rich domains (Fig. 2e), similar to the ‘nanogranite’ inclusions of Cesare *et al.*^30^. The mineral assemblage and modes in these domains are consistent with the fluxing melt being of intermediate (granodiorite) composition. In the final stages, pegmatitic garnet, plagioclase and hornblende crystallize (Fig. 1e).

Experimental research^31^ shows that hornblende, clinozoisite, biotite, plagioclase and granodioritic magma are stable between T = ~675–720 °C and 8–11 wt.% H_2_O at 8 kbar. These conditions correspond to temperature estimates of metamorphism in high-P shear zones in the valley^14^, suggesting the lower crust was at appropriate P-T conditions for a migrating granodioritic melt to dissolve plagioclase and pyroxene of the host rock via grain-scale magmatic assimilation and crystallize the observed mineral assemblage of the hornblendite bodies. The migration of intermediate composition magma through the lower crust of a magmatic arc is likely to be common.

Our model indicates the protolith to an ultrabasic rock, ubiquitous in the lower crust, does not necessarily have to form by cumulate processes or intrusion of unusually basic to ultrabasic magmas. These rocks may in fact be the geological expression of localized channels of melt flux in the lower crust. While initially reactive, these channels soon armor melt migration from chemical modification during global redistribution of matter through the crust and are likely to be important for transport of metals to sites of economic concentration.

The conclusion that an ultrabasic body can form by flux of an externally-derived melt and melt-rock interaction, rather than cumulate processes, invites a reevaluation of the significance of basic to ultrabasic bodies in exposures of lower crust, emphasizes their importance in delineating zones of mass transfer, and therefore may help resolve the cryptic pathways of melt migration at depth.

## Methods

Petrographic analysis of polished thin sections used a petrographic microscope in combination with the Virtual Petrographic Microscope^32^ and ImageJ v1.48a, along with back-scatter electron (BSE) imaging performed on a Carl Zeiss IVO scanning electron microscope (SEM; high vacuum, 30 kV accelerating voltage; Geochemical Analysis Unit, Macquarie University).

## Additional Information

**How to cite this article**: Daczko, N. R. *et al.* Hornblendite delineates zones of mass transfer through the lower crust. *Sci. Rep.* **6**, 31369; doi: 10.1038/srep31369 (2016).

## References

[b1] Bourdon E. *et al.* Adakite-like lavas from Antisana Volcano (Ecuador): Evidence for slab melt metasomatism beneath Andean Northern Volcanic Zone. Journal of Petrology 43, 199–217, doi: 10.1093/petrology/43.2.199 (2002).

[b2] Keay S., Collins W. J. & McCulloch M. T. A three-component Sr-Nd isotopic mixing model for granitoid genesis, Lachlan fold belt, eastern Australia. Geology 25, 307–310, doi: 10.1130/0091-7613(1997)025<0307:atcsni>2.3.co;2 (1997).

[b3] Rapp R. P., Shimizu N. & Norman M. D. Growth of early continental crust by partial melting of eclogite. Nature 425, 605–609, doi: 10.1038/nature02031 (2003). 14534583

[b4] Spence D. A., Sharp P. W. & Turcotte D. L. Buoyancy-driven crack propagation: a mechanism for magma migration. Journal of Fluid Mechanics 174, 135–153, doi: 10.1017/S0022112087000077 (1987).

[b5] Brown M. & Solar G. S. Shear-zone systems and melts: feedback relations and self-organization in orogenic belts. Journal of Structural Geology 20, 211–227, doi: 10.1016/S0191-8141(97)00068-0 (1998).

[b6] Rudnick R. L. Making continental crust. Nature 378, 571–578, doi: 10.1038/378571a0 (1995).

[b7] Clarke G. L., Klepeis K. A. & Daczko N. R. Cretaceous high-*P* granulites at Milford Sound, New Zealand: metamorphic history and emplacement in a convergent margin setting. Journal of Metamorphic Geology 18, 359–374, doi: 10.1046/j.1525-1314.2000.00259.x (2000).

[b8] Daczko N. R., Clarke G. L. & Klepeis K. A. Transformation of two-pyroxene hornblende granulite to garnet granulite involving simultaneous melting and fracturing of the lower crust, Fiordland, New Zealand. Journal of Metamorphic Geology 19, 549–562, doi: 10.1046/j.0263-4929.2001.00328.x (2001).

[b9] Allibone A. H. *et al.* Plutonic rocks of Western Fiordland, New Zealand: Field relations, geochemistry, correlation, and nomenclature. New Zealand Journal of Geology and Geophysics 52, 379–415, doi: 10.1080/00288306.2009.9518465 (2009).

[b10] Rosenberg C. L. & Riller U. Partial-melt topology in statically and dynamically recrystallized granite. Geology 28, 7–10, doi: 10.1130/0091-7613(2000)28<7:ptisad>2.0.co;2 (2000).

[b11] Holness M. B. & Sawyer E. W. On the pseudomorphing of melt-filled pores during the crystallization of migmatites. Journal of Petrology 49, 1343–1363, doi: 10.1093/petrology/egn028 (2008).

[b12] Holness M. B., Cesare B. & Sawyer E. W. Melted rocks under the microscope: Microstructures and their interpretations. Elements 7, 247–252, doi: 10.2113/gselements.7.4.247 (2011).

[b13] Smith J. R., Piazolo S., Daczko N. R. & Evans L. The effect of pre-tectonic reaction and annealing extent on behaviour during subsequent deformation: insights from paired shear zones in the lower crust of Fiordland, New Zealand. Journal of Metamorphic Geology 33, 557–577, doi: 10.1111/jmg.12132 (2015).

[b14] Daczko N. R., Klepeis K. A. & Clarke G. L. Evidence of Early Cretaceous collisional-style orogenesis in northern Fiordland, New Zealand and its effects on the evolution of the lower crust. Journal of Structural Geology 23, 693–713, doi: 10.1016/S0191-8141(00)00130-9 (2001).

[b15] Schröter F. C. *et al.* Trace element partitioning during high‐P partial melting and melt‐rock interaction; an example from northern Fiordland, New Zealand. Journal of Metamorphic Geology 22, 443–457, doi: 10.1111/j.1525-1314.2004.00525.x (2004).

[b16] Smith D. J. Clinopyroxene precursors to amphibole sponge in arc crust. Nature Communications 5, doi: 10.1038/ncomms5329 (2014). PMC4102121 25002269

[b17] Dijkstra A. H., Barth M. G., Drury M. R., Mason P. R. D. & Vissers R. L. M. Diffuse porous melt flow and melt‐rock reaction in the mantle lithosphere at a slow‐spreading ridge: A structural petrology and LA‐ICP‐MS study of the Othris Peridotite Massif (Greece). Geochemistry, Geophysics, Geosystems 4, doi: 10.1029/2001GC000278 (2003).

[b18] Jagoutz O., Müntener O., Burg J. P., Ulmer P. & Jagoutz E. Lower continental crust formation through focused flow in km-scale melt conduits: The zoned ultramafic bodies of the Chilas Complex in the Kohistan island arc (NW Pakistan). Earth and Planetary Science Letters 242, 320–342, doi: 10.1016/j.epsl.2005.12.005 (2006).

[b19] Kelemen P. B., Shimizu N. & Salters V. J. M. Extraction of mid-ocean-ridge basalt from the upwelling mantle by focused flow of melt in dunite channels. Nature 375, 747–753, doi: 10.1038/375747a0 (1995).

[b20] Fusseis F., Regenauer-Lieb K., Liu J., Hough R. M. & De Carlo F. Creep cavitation can establish a dynamic granular fluid pump in ductile shear zones. Nature 459, 974–977, doi: 10.1038/nature08051 (2009). 19536262

[b21] Hasalová P. *et al.* Origin of migmatites by deformation-enhanced melt infiltration of orthogneiss: a new model based on quantitative microstructural analysis. Journal of Metamorphic Geology 26, 29–53, doi: 10.1111/j.1525-1314.2007.00743.x (2008).

[b22] Schulmann K. *et al.* Evolution of microstructure and melt topology in partially molten granitic mylonite: Implications for rheology of felsic middle crust. Journal of Geophysical Research: Solid Earth 113, n/a-n/a, doi: 10.1029/2007JB005508 (2008).

[b23] Katz R. F., Spiegelman M. & Holtzman B. The dynamics of melt and shear localization in partially molten aggregates. Nature 442, 676–679, doi: 10.1038/nature05039 (2006). 16900197

[b24] Kelemen P. B., Dick H. J. B. & Quick J. E. Formation of harzburgite by pervasive melt/rock reaction in the upper mantle. Nature 358, 635–641, doi: 10.1038/358635a0 (1992).

[b25] Daines M. J. & Kohlstedt D. L. The transition from porous to channelized flow due to melt/rock reaction during melt migration. Geophysical Research Letters 21, 145–148, doi: 10.1029/93GL03052 (1994).

[b26] Pec M., Holtzman B. K., Zimmerman M. & Kohlstedt D. L. Reaction infiltration instabilities in experiments on partially molten mantle rocks. Geology 43, 575–578, doi: 10.1130/g36611.1 (2015).

[b27] Spiegelman M., Kelemen P. B. & Aharonov E. Causes and consequences of flow organization during melt transport: The reaction infiltration instability in compactible media. Journal of Geophysical Research 106, 2061–2077, doi: 10.1029/2000JB900240 (2001).

[b28] Connolly J. A. D. & Podladchikov Y. Y. Decompaction weakening and channeling instability in ductile porous media: Implications for asthenospheric melt segregation. Journal of Geophysical Research: Solid Earth 112, B10205, doi: 10.1029/2005JB004213 (2007).

[b29] Spiegelman M. Flow in deformable porous media. Part 2. Numerical analysis-the relationship between shock waves and solitary waves. Journal of Fluid Mechanics 247, 39–39, doi: 10.1017/S0022112093000370 (1993).

[b30] Cesare B., Ferrero S., Salvioli-Mariani E., Pedron D. & Cavallo A. “Nanogranite” and glassy inclusions: The anatectic melt in migmatites and granulites. Geology 37, 627–630, doi: 10.1130/g25759a.1 (2009).

[b31] Naney M. T. Phase equilibria of rock-forming ferromagnesian silicates in granitic systems. American Journal of Science 283, 993–1033, doi: 10.2475/ajs.283.10.993 (1983).

[b32] Tetley M. G. & Daczko N. R. Virtual Petrographic Microscope: a multi-platform education and research software tool to analyse rock thin-sections. Australian Journal of Earth Sciences 61, 631–637, doi: 10.1080/08120099.2014.886624 (2014).

